# Plant-based diets in patients with chronic kidney disease

**DOI:** 10.2478/abm-2024-0002

**Published:** 2024-03-20

**Authors:** Wannasit Wathanavasin, Piyawan Kittiskulnam, Kirsten L. Johansen

**Affiliations:** Nephrology Unit, Department of Medicine, Charoenkrung Pracharak Hospital, Bangkok Metropolitan Administration, Bangkok 10330, Thailand; Division of Nephrology, Department of Medicine, Faculty of Medicine, Chulalongkorn University, Bangkok 10330, Thailand; Division of Internal Medicine-Nephrology, Department of Medicine, Faculty of Medicine, King Chulalongkorn Memorial Hospital, Thai Red Cross Society, Bangkok 10330, Thailand; Special Task Force for Activating Research in Renal Nutrition (Renal Nutrition Research Group), Office of Research Affairs, Chulalongkorn University, Bangkok 10330, Thailand; Division of Nephrology, Hennepin Healthcare, Minneapolis, MN 55415, USA; Division of Nephrology, University of Minnesota, Minneapolis, MN 55415, USA

**Keywords:** chronic kidney disease, low-protein diet, nutrition, plant-based diets

## Abstract

Dietary protein restriction has been considered to be a nutritional-related strategy to reduce risk for end-stage kidney disease among patients with non-dialysis-dependent chronic kidney disease (CKD). However, there is insufficient evidence to recommend a particular type of protein to slow down the CKD progression. Recently, various plant-based diets could demonstrate some additional benefits such as a blood pressure-lowering effect, a reduction of metabolic acidosis as well as hyperphosphatemia, and gut-derived uremic toxins. Furthermore, the former concerns about the risk of undernutrition and hyperkalemia observed with plant-based diets may be inconsistent in real clinical practice. In this review, we summarize the current evidence of the proposed pleiotropic effects of plant-based diets and their associations with clinical outcomes among pre-dialysis CKD patients.

Chronic kidney disease (CKD) remains a global public health concern that affects around 10% of the general population worldwide [1]. As a consequence of impaired kidney function, patients with CKD are at higher risk of unfavorable outcomes, including cardiovascular disease-related death and all-cause mortality, and risk is particularly high among those with advanced CKD [2]. Therefore, slowing CKD progression has the potential to lower risk of death and to prolong the time to end-stage kidney disease (ESKD) or kidney replacement therapy (KRT) [3]. Dietary protein restriction has long been a mainstay of nutrition therapy to retard CKD progression by reducing urinary protein excretion and glomerular hyperfiltration [4]. According to the National Kidney Foundation/Kidney Disease Outcomes Quality Initiative (NKF/KDOQI) 2000 nutrition guideline in CKD [5], lowering protein intake to 0.6 g/kg/d was recommended for patients with an estimated glomerular filtration rate (eGFR) of <25 mL/min/1.73 m^2^ to retard the progression of CKD. This guideline also mentioned that >50% of protein intake should be of high biological value, which is likely to be an animal protein in origin. However, the statement regarding a particular protein type has not been fully addressed by the updated KDOQI guideline in 2020 [6] due to insufficient evidence.

The interest in plant-based diets has increased over time because of their potential cardiovascular health benefits, such as the reduction of incidence of ischemic heart disease [7] and cardiovascular mortality [8] among the general population. In addition, plant-based diets demonstrate a favorable impact on kidney outcomes in patients with CKD due to their utility for managing CKD-related complications, including metabolic acidosis, hyperphosphatemia, and hypertension. This review article aims to focus on the use of plant-based diets in patients with pre-dialysis CKD and highlight their pleiotropic effects and potential concerns in clinical practice.

## Different types of protein diet and kidney outcomes

Several previous studies revealed that a high protein intake increased the risk of glomerular hyperfiltration and had deleterious effects on kidney function in both the general population [9, 10, 11] and patients with pre-existing CKD [12, 13]. A high protein consumption leads to multiple abnormalities in renal hemodynamics, primarily from the vasodilation of afferent arteriole, followed by an increase in intraglomerular pressure, which results in proteinuria and glomerulosclerosis [14]. At the cellular level, the effect of glomerular hyperfiltration may also promote abnormal mesangial cell signaling and overexpression of transforming growth factor β (TGF-β), causing interstitial inflammation and fibrosis [15]. Furthermore, consuming excessive dietary protein has been linked to metabolic disorders, including metabolic acidosis [16, 17] and hyperphosphatemia [18]. Although the findings from the “Modification of Diet in Renal Disease” (MDRD) study failed to demonstrate effectiveness of a low protein diet (LPD) in retarding CKD progression over a 2-year duration [12], a reanalysis of that study suggested that the LPD demonstrated a reno-protective effect after implementation for a longer period of time [19]. Additionally, most well-controlled trials [20, 21] and meta-analyses [22, 23, 24] have consistently supported a benefit of dietary protein restriction on slowing CKD progression. Based on the aforementioned evidence, the updated KDOQI guidelines stated that “a protein-restricted diet strategy is recommended in stages 3–5 CKD.” A LPD, approximately 0.6–0.8 g/kg/d, is strongly recommended in metabolically stable patients with diabetic and non-diabetic CKD to reduce the risk of ESKD or death. Besides the quantity of protein consumed, awareness of its quality (animal or plant) has increased among CKD patients. However, the guideline did not address protein type due to insufficient evidence.

Earlier observational studies [25, 26, 27, 28] have shown a positive correlation between animal-based diets, especially red meat consumption [29], and the development of new-onset proteinuria and incident CKD in the general population. A study by Kontessis et al. [30] revealed that consuming diets high in animal-based protein in healthy subjects led to a glomerular hyperfiltration-related higher level of proteinuria and eGFR than plant-based protein diets at the same amount of total daily protein intake. In their study, an acute protein load of meat consumption was significantly associated with an increase in fractional albumin clearance and renal plasma flow by 40% and 14%, respectively. In the Nurses' Health Study (NHS) [31], a high intake of non-dairy animal protein in participants with CKD and an eGFR between 50 mL/min/1.73 m^2^ and 88 mL/min/1.73 m^2^ were also significantly associated with a faster decline in eGFR of 1.2 mL/min/1.73 m^2^ [95% confident interval, CI; –2.3 to –0.3] per 10-g increase in animal protein intake. Likewise, several controlled trials [32, 33] revealed that partial replacement of animal protein with plant protein among patients with CKD receiving angiotensin-converting enzyme inhibitors additionally reduced the degree of albuminuria by average 10%–20% when compared with baseline values in the replacement group with plant-based protein.

The ability of plant-based proteins to meet human nutrition requirements has been debated when compared with animal-based proteins. Emerging evidence indicates that the conventional definition of a high-biological protein is constrained. To measure protein quality, the protein digestibility–corrected amino acid score (PDCAAS) and digestible indispensable amino acid score (DIAAS) adopted by the Food and Agricultural Organization of the United Nations and World Health Organization (FAO/WHO) are currently the preferred methods. PDCAAS and DIAAS are used to measure the protein digestible quality based on both human amino acid requirements and digestibility quality. A PDCAAS score <0.75 indicates that the protein is suboptimal, and a DIAAS score >1.0 denotes that there may be an increased benefit for health [34, 35].

Animal-based proteins appear to have both higher PDCAAS and DIAAS scores (>0.9) compared with plant-based proteins, which range from 0.4 to 0.9, as shown in **Table 1**. However, with an adequate diet and blending the proper combination, consuming protein from plants does not increase the risk of malnutrition [36]. According to a meta-analysis by Rand et al. [37], healthy adults can meet their protein needs regardless of dietary protein sources, whether animal, vegetable, or mixed. Furthermore, data from real-world studies [38, 39] also supported and emphasized that a concept of well-balanced and diversified plant-based diets, focusing on a high proportion of plant proteins and complex carbohydrates, such as quinoa, tofu, grains, soya, legumes, sweet potatoes, and brown rice, is nutritionally sufficient without amino acid deficiency. A study by Barsotti et al. [40] illustrated that patients with CKD treated with a low-protein diet of 0.7 g/kg/d with mixed plant-protein content, particularly cereals and legumes, could reach minimal requirements for essential amino acids, and none of the patients had nutritional deficiency after a 1-year follow-up.

**Table 1.** PDCAAS/DIAAS for animal- vs plant-based isolated protein and food

**Food**	**PDCAAS**	**DIAAS**	**Limiting amino acids**
Animal-based protein			
Milk protein concentrate	1.00	1.18	Met + Cys
Whole milk	1.00	1.14	Met + Cys
Egg (hard boiled)	1.00	1.13	His
Chicken breast	1.00	1.08	Trp
Ground Beef (cooked)	0.92	0.99	Leu
Plant-based protein			
Soy protein isolate	0.98	0.90	Met + Cys
Pea protein isolate	0.89	0.82	Met + Cys
Cooked rice	0.62	0.59	Lys
Tofu	0.70	0.97	Met + Cys

Adapted from Ref. [41, 42, 43, 44].

Cys, cysteine; DIAAS, digestible indispensable amino acid score; His, histidine; Lys, Lysine; Met, methionine; PDCAAS, protein digestibility corrected amino acid score; Trp, tryptophan.

### Spectrum of plant-based dietary patterns and their benefits in CKD

**Figure 1.**
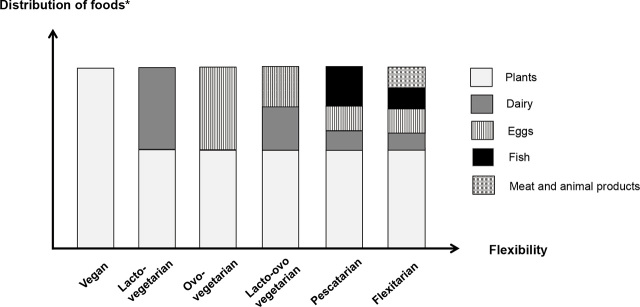
The plant-based dietary spectrum. From left (less flexible) to right (more flexible): vegan diet (including only plant-based items), lacto-vegetarian diet (excluding meat, fish, or poultry but including dairy), ovo-vegetarian diet (excluding meat, fish, or poultry but including eggs), lacto-ovo-vegetarian diet (excluding meat, fish, or poultry but including dairy and eggs), pescatarian diet (excluding meat or poultry but including fish) and omnivorous diet (containing all food groups).

In general, plant-based diets include high proportions of vegetables, grains, legumes, fruits, and nuts, with a reduction or elimination of animal product consumption (**Figure 1**). Whole, unprocessed plant-based foods are preferred to their highly processed forms.

Among patients with non-dialysis-dependent CKD, the PLAnt-DOminant low-protein diet (PLADO) has recently been proposed by Kalantar-Zadeh et al. [45]. PLADO is defined as an LPD with at least 50% of protein from plants, preferably whole, unrefined, and unprocessed foods. Additional characteristics of PLADO include a relatively low consumption of salt (<3 g/d), a greater intake of dietary fiber (at least 25–30 g/d), and a sufficient intake of calories (30–35 kcal/kg/d). There are several putative mechanisms by which patients with CKD would benefit from plant-based diets, as shown in **Table 2**.

**Table 2.** Overview of plant vs animal foods for management of CKD-related complications

**CKD-related complications**	**Animal foods**	**Plant foods**
Metabolic acidosis	Worsen	Improve
	(high non-volatile acid load)	(high contents of citrate and malate)
Hyperphosphatemia	Worsen	Improve
	(high phosphate bioavailability)	(low phosphate bioavailability)
Uremic toxins	Likely worsen	Likely improve
	(harbors proteolytic bacteria)	(harbors saccharolytic bacteria)
Inflammation	Likely worsen	Likely improve
	(low antioxidants and no fiber)	(high antioxidants and fiber)
Hypertension	-	Improve
		(high potassium and nitrate content)
Hyperkalemia	Less likely to cause hyperkalemia	Less likely to cause hyperkalemia
	(usually low K^+^ contents if unprocessed foods)	(high K^+^ contents but increase bowel movement and K^+^ excretion)

K^+^, potassium; -, denotes a lack of sufficient evidence of their associations.

CKD, chronic kidney disease.

#### Metabolic acidosis

Unlike animal products, plant-based foods provide natural alkali in the form of citrate and malate, which can be converted to bicarbonate. Studies from Goraya and colleagues [46, 47] supported that the correction of metabolic acidosis with administration of oral sodium bicarbonate or alkali-producing fruits and vegetables (FV) attenuated kidney injury in patients with hypertensive nephropathy. The amelioration of acid retention is partly explained by a reduction in tubulo-interstitial injury, as demonstrated by a decrease in urine TGF-β level. As compared with oral sodium bicarbonate tablets, dietary acid reduction with plant-based diets additionally reduced dietary sodium intake from alkali therapy, resulting in lower arterial blood pressure, and also promoted weight reduction after a 3-year follow-up period [47].

#### Hyperphosphatemia and elevated fibroblast growth factor 23 (FGF-23) level

Although most plant foods contain higher levels of phosphorus than animal foods, intestinal absorption of phosphate from plant sources, which is mostly in the form of phytate, does not exceed 50% due to the absence of human phytase activity [18]. Moreover, the phosphate bioavailability of animal foods is typically higher (40%–80%) than that of plant foods, particularly when phosphorus-based preservatives are used for meat processing [48]. In addition, similar results reported by Moe et al. [49] and Di Iorio et al. [50] showed that a vegetarian diet was associated with a significant reduction in serum phosphorus and FGF-23 levels in patients with CKD.

#### Gut dysbiosis and its uremic toxins

Plants are the source of dietary fiber that preferentially promotes the proliferation of saccharolytic bacteria. These bacteria can break down fiber and produce short-chain fatty acids (e.g., acetate, butyrate, and propionate), which improve epithelial barrier integrity and lessen bacterial translocation and inflammation. By contrast, animal-based protein diets appear to generate a nitrogen-rich environment and promote the absorption of gut-derived uremic toxins. Notably, higher intake of animal-based foods in omnivorous diets has been linked to higher levels of circulating trimethylamine N-oxide (TMAO). Recent evidence reveals that this small molecule uremic toxin promotes the acceleration of atherosclerosis [51]. A study of McFarlane et al. [52] demonstrated that in patients with CKD stages 3–4, a higher intake of healthy plant foods (high plant-based diet index [PDI]; a measure of adherence to a high-quality plant-based diet) and a habitual intake of dietary fiber resulted in lower serum levels of free p-cresyl sulfate and indoxyl sulfate. These two prototypes of protein-bound uremic toxins were associated with an increased risk of cardiovascular events and mortality in CKD patients [53, 54, 55]. It is likely that consumption of a plant-based, fiber-rich dietary pattern may result in beneficial changes in the gut microbiota, which lead to better clinical outcomes in patients with CKD.

#### Hypertension

There are several possible explanations for the blood pressure reduction in plant-based foods. Plant-based diets, particularly vegetarian diets, have a beneficial effect on weight reduction and low rates of overweight and obesity due to their relatively high fiber, reduced fat, and low energy content [56, 57]. A meta-analysis of controlled trials by Neter et al. [58] revealed that an average reduction of 5 kg of body weight significantly decreased systolic blood pressure by 4.4 mmHg. In particular, FV are the main sources of potassium, nitrate, and fiber. It is hypothesized that high potassium and nitrate intake promotes vasodilation and improves endothelial function while inhibiting reactive oxygen species production and platelet aggregation [59, 60]. Also, a report by Tuttle et al. [61] indicated that patients with a preference for plant-based diets had high serum levels of histidine and threonine, which have been reported to be associated with an improvement in blood pressure control by decreasing central sympathetic output and increasing nitric oxide in the brain vasomotor center [62].

#### CKD progression

An analysis using data from the Third National Health and Nutrition Examination Survey (NHANES) suggested that a higher intake of FV, assessed by the food frequency questionnaire (FFQ), was associated with a lower risk of ESKD requiring KRT among patients with CKD [63]. Compared with the highest quintile (eating FV 6 times/d), eating FV <2 times/d was associated with a statistically significant 41% higher risk of ESKD. Similarly, Lew et al. [29] revealed that red meat consumption was dose-dependently associated with higher risk of kidney failure, whereas substituting one serving of red meat with soy or legumes was associated with a 50.4% reduction in the risk of ESKD. A study by Dinu et al. [64] in a CKD population also showed that those consuming a lactoovo vegetarian diet had a significantly higher eGFR with a mean difference of 4.2 mL/min/1.73 m^2^ compared with those following the meat and fish-containing Mediterranean diet. Data from the Atherosclerosis Risk in Communities (ARIC) study among a community-based cohort of 15,792 middle-aged adults [65] also reported that higher adherence to a healthy plant-based diet, particularly in the highest quintile of diet quality, assessed by a modified semiquantitative FFQ was associated with a slower annual eGFR decline (−1.46; 95% CI, −1.50 to −1.43 mL/min/1.73 m^2^) compared with those in the lowest quintile of diet quality (−1.57; 95% CI, −1.6 to −1.43 mL/min/1.73 m^2^; *P* for trend = 0.001). To assess the association between the source of dietary protein and eGFR decline, the Longitudinal Study of Ageing Women cohort [66] reported that higher intake of plant-sourced protein was associated with a slower decline in kidney function after adjusting for confounders including animal protein and energy intake. Each 10-g increase in protein intake from plants was associated with a reduction in the annual eGFR decline (0.12; 95% CI, 0.01–0.23) mL/min/1.73 m^2^), whereas intake of animal-sourced protein was not associated with retardation of eGFR decline (0.01; 95% CI, −0.04 to 0.05 mL/min/1.73 m^2^ per a 10-g increase in animal protein).

### Potential concerns of plant-based diets in CKD

#### Nutrient deficiency

Previous studies demonstrated that consumption of a plant-based, LPD with adequate calories and a variety of food groups did not increase the risk of protein energy wasting, monitored by body weight and serum biochemistry, in stable patients with CKD [45, 67, 68, 69]. Because they maintained neutral nitrogen balance with sufficient calorie supply, these individuals maintained normal anthropometric estimates of lean body mass and nutritional status regardless of plant-or animal-based food types. With regard to micronutrients, Neufingerl and Eilander. [70] found that healthy individuals following self-selected plant-based diets, especially vegan diets, for a duration of several years increased their risk of inadequacy of some essential micronutrients such as vitamin B12, vitamin D, iron, zinc, and iodine. Thus, careful planning with frequent monitoring and assessment of nutritional status is required for long-term consumption of plant-based diets, particularly vegetarian diets. In addition, education on diverse nutrient-dense plant foods, food fortification, and possibly supplementation should be individually emphasized.

#### Hyperkalemia

**Figure 2.**
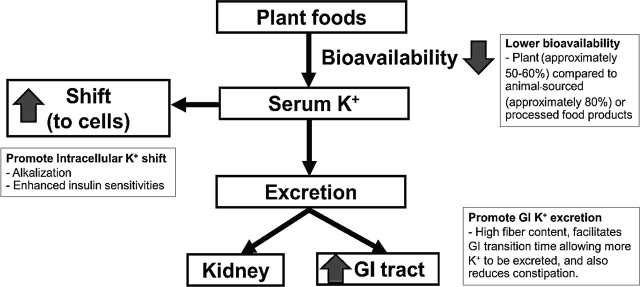
Effects of plant foods on serum potassium level, including potassium bioavailability, shift and loss. GI, gastrointestinal; K^+^, potassium.

Some evidence indicates that plant-based diets are not associated with hyperkalemia in most patients with CKD, particularly CKD stages 3 and 4 [40, 71, 72, 73, 74]. Concentrated potassium content (e.g., juice, sauce, and dried fruit), food additives, and preservatives, but not whole-food forms, are the main hidden sources of dietary potassium intake [75, 76]. It should be recognized that muscle-based animal products, such as meat and poultry, are also naturally high in potassium content. To reduce the potassium content, boiling and soaking (and discarding the water) are effective cooking methods, leading to a loss of 60%–80% of the potassium in several raw foods [77]. Besides dietary potassium load, there are several factors, including potassium bioavailability, shift, and excretion, which affect serum potassium levels (**Figure 2**). Plant-based foods, particularly in their whole and unprocessed form, tend to have a lower potassium bioavailability (approximately 50%–60%) compared with animal-sourced (approximately 80%) or processed food products (nearly 100%) [67]. Alkalization due to the presence of base-producing organic anions, including malate and citrate [78], combined with the enhanced insulin sensitivity [79] associated with plant-based dietary patterns may facilitate intracellular potassium shifts [80]. Due to its high fiber content, a plant-based diet facilitates gastrointestinal transition time, allowing more potassium to be excreted, and also reduces constipation [81]. For these reasons, the risk of hyperkalemia from plant-based diets may have been previously overstated. Indeed, not all plant-based foods have the same potential to cause elevated serum potassium levels.

**Figure 3.**
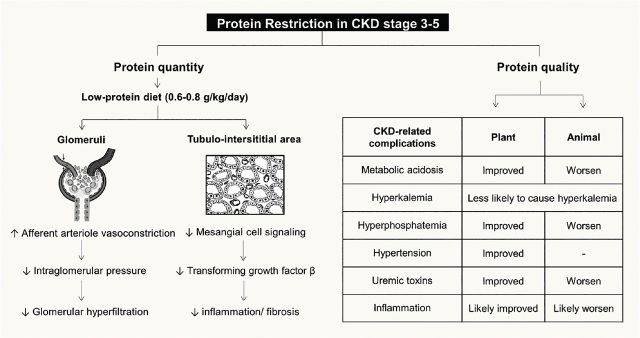
Concept of protein restriction in CKD stages 3–5, focusing on both protein quantity, low-protein diet (0.6–0.8 g/kg/d) in order to slow down the CKD progression, and protein quality, plant-compared to animal-based protein in managing CKD-related complications. Adapted from [82]. CKD, chronic kidney disease.

## Conclusion

According to the principles of “precision nutrition,” not all patients with CKD should be routinely prescribed a plant-based diet. Implementation of a plant-based diet as an option for patients with CKD for managing CKD-related complications and slowing CKD progression requires agreement between patient and health-care provider team, including at least a nephrologist and renal dietitian. A more flexible strategy, based on the patient's habits and preferences, tends to achieve better results. Furthermore, patient communication and motivation are crucial to improve their adherence. Patients should be counseled on eating well-balanced and diversified plant-based diets to achieve nutritional sufficiency. Further well-controlled randomized studies are needed to address knowledge gaps for the implementation of plant-based diets in real clinical practice.

## References

[1] Kovesdy CP (2022). Epidemiology of chronic kidney disease: an update 2022. Kidney Int Suppl.

[2] Jankowski J, Floege J, Fliser D, Böhm M, Marx N (2021). Cardiovascular disease in chronic kidney disease: pathophysiological insights and therapeutic options. Circulation.

[3] Turner JM, Bauer C, Abramowitz MK, Melamed ML, Hostetter TH (2012). Treatment of chronic kidney disease. Kidney Int.

[4] Ruilope LM, Casal MC, Praga M, Alcazar JM, Decap G, Lahera V, Rodicio JL (1992). Additive antiproteinuric effect of converting enzyme inhibition and a low protein intake. J Am Soc Nephrol.

[5] (2000). Clinical practice guidelines for nutrition in chronic renal failure. K/DOQI, National Kidney Foundation. Am J Kidney Dis.

[6] Ikizler TA, Burrowes JD, Byham-Gray LD, Campbell KL, Carrero JJ, Chan W (2020). KDOQI clinical practice guideline for nutrition in CKD: 2020 update. Am J Kidney Dis.

[7] Crowe FL, Appleby PN, Travis RC, Key TJ (2013). Risk of hospitalization or death from ischemic heart disease among British vegetarians and nonvegetarians: results from the EPIC-Oxford cohort study. Am J Clin Nutr.

[8] Kim H, Caulfield LE, Garcia-Larsen V, Steffen LM, Coresh J, Rebholz CM (2019). Plant-based diets are associated with a lower risk of incident cardiovascular disease, cardiovascular disease mortality, and all-cause mortality in a general population of middle-aged adults. J Am Heart Assoc.

[9] Friedman AN (2004). High-protein diets: potential effects on the kidney in renal health and disease. Am J Kidney Dis.

[10] Frank H, Graf J, Amann-Gassner U, Bratke R, Daniel H, Heemann U, Hauner H (2009). Effect of short-term high-protein compared with normal-protein diets on renal hemodynamics and associated variables in healthy young men. Am J Clin Nutr.

[11] Jhee JH, Kee YK, Park S, Kim H, Park JT, Han SH (2020). High-protein diet with renal hyperfiltration is associated with rapid decline rate of renal function: a community-based prospective cohort study. Nephrol Dial Transplant.

[12] Klahr S, Levey AS, Beck GJ, Caggiula AW, Hunsicker L, Kusek JW, Striker G (1994). The effects of dietary protein restriction and blood-pressure control on the progression of chronic renal disease. Modification of Diet in Renal Disease Study Group. N Engl J Med.

[13] Levey AS, Adler S, Caggiula AW, England BK, Greene T, Hunsicker LG (1996). Effects of dietary protein restriction on the progression of advanced renal disease in the Modification of Diet in Renal Disease Study. Am J Kidney Dis.

[14] Kramer H (2017). Kidney disease and the westernization and industrialization of food. Am J Kidney Dis.

[15] Kalantar-Zadeh K, Fouque D (2017). Nutritional management of chronic kidney disease. N Engl J Med.

[16] Mardon J, Habauzit V, Trzeciakiewicz A, Davicco MJ, Lebecque P, Mercier S (2008). Long-term intake of a high-protein diet with or without potassium citrate modulates acid-base metabolism, but not bone status, in male rats. J Nutr.

[17] Remer T (2001). Influence of nutrition on acid-base balance – metabolic aspects. Eur J Nutr.

[18] Kalantar-Zadeh K, Gutekunst L, Mehrotra R, Kovesdy CP, Bross R, Shinaberger CS (2010). Understanding sources of dietary phosphorus in the treatment of patients with chronic kidney disease. Clin J Am Soc Nephrol.

[19] Levey AS, Greene T, Sarnak MJ, Wang X, Beck GJ, Kusek JW (2006). Effect of dietary protein restriction on the progression of kidney disease: long-term follow-up of the modification of diet in renal disease (MDRD) study. Am J Kidney Dis.

[20] Ihle BU, Becker GJ, Whitworth JA, Charlwood RA, Kincaid-Smith PS (1989). The effect of protein restriction on the progression of renal insufficiency. N Engl J Med.

[21] Garneata L, Stancu A, Dragomir D, Stefan G, Mircescu G (2016). Ketoanalogue-supplemented vegetarian very low-protein diet and CKD progression. J Am Soc Nephrol.

[22] Yan B, Su X, Xu B, Qiao X, Wang L (2018). Effect of diet protein restriction on progression of chronic kidney disease: a systematic review and meta-analysis. PLoS One.

[23] Rhee CM, Ahmadi SF, Kovesdy CP, Kalantar-Zadeh K (2018). Low-protein diet for conservative management of chronic kidney disease: a systematic review and meta-analysis of controlled trials. J Cachexia Sarcopenia Muscle.

[24] Nezu U, Kamiyama H, Kondo Y, Sakuma M, Morimoto T, Ueda S (2013). Effect of low-protein diet on kidney function in diabetic nephropathy: meta-analysis of randomised controlled trials. BMJ Open.

[25] Yuzbashian E, Asghari G, Mirmiran P, Hosseini FS, Azizi F (2015). Associations of dietary macronutrients with glomerular filtration rate and kidney dysfunction: Tehran lipid and glucose study. J Nephrol.

[26] Haring B, Selvin E, Liang M, Coresh J, Grams ME, Petruski-Ivleva N (2017). Dietary protein sources and risk for incident chronic kidney disease: results from the Atherosclerosis Risk in Communities (ARIC) study. J Ren Nutr.

[27] Lin J, Hu FB, Curhan GC (2010). Associations of diet with albuminuria and kidney function decline. Clin J Am Soc Nephrol.

[28] Mirmiran P, Yuzbashian E, Aghayan M, Mahdavi M, Asghari G, Azizi F (2020). A prospective study of dietary meat intake and risk of incident chronic kidney disease. J Ren Nutr.

[29] Lew QJ, Jafar TH, Koh HW, Jin A, Chow KY, Yuan JM, Koh WP (2017). Red meat intake and risk of ESRD. J Am Soc Nephrol.

[30] Kontessis P, Jones S, Dodds R, Trevisan R, Nosadini R, Fioretto P (1990). Renal, metabolic and hormonal responses to ingestion of animal and vegetable proteins. Kidney Int.

[31] Knight EL, Stampfer MJ, Hankinson SE, Spiegelman D, Curhan GC (2003). The impact of protein intake on renal function decline in women with normal renal function or mild renal insufficiency. Ann Intern Med.

[32] Teixeira SR, Tappenden KA, Carson L, Jones R, Prabhudesai M, Marshall WP, Erdman JW (2004). Isolated soy protein consumption reduces urinary albumin excretion and improves the serum lipid profile in men with type 2 diabetes mellitus and nephropathy. J Nutr.

[33] Azadbakht L, Atabak S, Esmaillzadeh A (2008). Soy protein intake, cardiorenal indices, and C-reactive protein in type 2 diabetes with nephropathy: a longitudinal randomized clinical trial. Diabetes Care.

[34] Rojas Conzuelo Z, Bez NS, Theobald S, Kopf-Bolanz KA (2022). Protein quality changes of vegan day menus with different plant protein source compositions. Nutrients.

[35] Mathai JK, Liu Y, Stein HH (2017). Values for digestible indispensable amino acid scores (DIAAS) for some dairy and plant proteins may better describe protein quality than values calculated using the concept for protein digestibility-corrected amino acid scores (PDCAAS). Br J Nutr.

[36] Hertzler SR, Lieblein-Boff JC, Weiler M, Allgeier C (2020). Plant proteins: assessing their nutritional quality and effects on health and physical function. Nutrients.

[37] Rand WM, Pellett PL, Young VR (2003). Meta-analysis of nitrogen balance studies for estimating protein requirements in healthy adults. Am J Clin Nutr.

[38] Rosell M, Appleby P, Key T (2005). Height, age at menarche, body weight and body mass index in life-long vegetarians. Public Health Nutr.

[39] Appleby PN, Thorogood M, Mann JI, Key TJ (1999). The Oxford vegetarian study: an overview. Am J Clin Nutr.

[40] Barsotti G, Morelli E, Cupisti A, Meola M, Dani L, Giovannetti S (1996). A low-nitrogen low-phosphorus Vegan diet for patients with chronic renal failure. Nephron.

[41] Phillips SM (2017). Current concepts and unresolved questions in dietary protein requirements and supplements in adults. Front Nutr.

[42] Rutherfurd SM, Fanning AC, Miller BJ, Moughan PJ (2015). Protein digestibility-corrected amino acid scores and digestible indispensable amino acid scores differentially describe protein quality in growing male rats. J Nutr.

[43] (2013). Dietary protein quality evaluation in human nutrition. Report of an FAQ Expert Consultation. FAO Food Nutr Pap.

[44] Bailey HM, Mathai JK, Berg EP, Stein HH (2020). Most meat products have digestible indispensable amino acid scores that are greater than 100, but processing may increase or reduce protein quality. Br J Nutr.

[45] Kalantar-Zadeh K, Joshi S, Schlueter R, Cooke J, Brown-Tortorici A, Donnelly M (2020). Plant-dominant low-protein diet for conservative management of chronic kidney disease. Nutrients.

[46] Goraya N, Simoni J, Jo C, Wesson DE (2012). Dietary acid reduction with fruits and vegetables or bicarbonate attenuates kidney injury in patients with a moderately reduced glomerular filtration rate due to hypertensive nephropathy. Kidney Int.

[47] Goraya N, Simoni J, Jo CH, Wesson DE (2014). Treatment of metabolic acidosis in patients with stage 3 chronic kidney disease with fruits and vegetables or oral bicarbonate reduces urine angiotensinogen and preserves glomerular filtration rate. Kidney Int.

[48] Sherman RA, Mehta O (2009). Phosphorus and potassium content of enhanced meat and poultry products: implications for patients who receive dialysis. Clin J Am Soc Nephrol.

[49] Moe SM, Zidehsarai MP, Chambers MA, Jackman LA, Radcliffe JS, Trevino LL (2011). Vegetarian compared with meat dietary protein source and phosphorus homeostasis in chronic kidney disease. Clin J Am Soc Nephrol.

[50] Di Iorio B, Di Micco L, Torraca S, Sirico ML, Russo L, Pota A (2012). Acute effects of very-low-protein diet on FGF23 levels: a randomized study. Clin J Am Soc Nephrol.

[51] Zhu Y, Li Q, Jiang H (2020). Gut microbiota in atherosclerosis: focus on trimethylamine N-oxide. APMIS.

[52] McFarlane C, Krishnasamy R, Stanton T, Savill E, Snelson M, Mihala G (2022). Diet quality and protein-bound uraemic toxins: investigation of novel risk factors and the role of microbiome in chronic kidney disease. J Ren Nutr.

[53] Lin CJ, Wu V, Wu PC, Wu CJ (2015). Meta-analysis of the associations of p-cresyl sulfate (PCS) and indoxyl sulfate (IS) with cardiovascular events and all-cause mortality in patients with chronic renal failure. PLoS One.

[54] Liabeuf S, Barreto DV, Barreto FC, Meert N, Glorieux G, Schepers E (2010). Free p-cresylsulphate is a predictor of mortality in patients at different stages of chronic kidney disease. Nephrol Dial Transplant.

[55] Barreto FC, Barreto DV, Liabeuf S, Meert N, Glorieux G, Temmar M (2009). Serum indoxyl sulfate is associated with vascular disease and mortality in chronic kidney disease patients. Clin J Am Soc Nephrol.

[56] Fraser GE (2009). Vegetarian diets: what do we know of their effects on common chronic diseases?. Am J Clin Nutr..

[57] Barnard ND, Levin SM, Yokoyama Y (2015). A systematic review and meta-analysis of changes in body weight in clinical trials of vegetarian diets. J Acad Nutr Diet.

[58] Neter JE, Stam BE, Kok FJ, Grobbee DE, Geleijnse JM (2003). Influence of weight reduction on blood pressure: a meta-analysis of randomized controlled trials. Hypertension.

[59] Hobbs DA, George TW, Lovegrove JA (2013). The effects of dietary nitrate on blood pressure and endothelial function: a review of human intervention studies. Nutr Res Rev.

[60] McDonough AA, Nguyen MT (2012). How does potassium supplementation lower blood pressure?. Am J Physiol Renal Physiol..

[61] Tuttle KR, Milton JE, Packard DP, Shuler LA, Short RA (2012). Dietary amino acids and blood pressure: a cohort study of patients with cardiovascular disease. Am J Kidney Dis.

[62] Toba H, Nakamori A, Tanaka Y, Yukiya R, Tatsuoka K, Narutaki M (2010). Oral L-histidine exerts antihypertensive effects via central histamine H3 receptors and decreases nitric oxide content in the rostral ventrolateral medulla in spontaneously hypertensive rats. Clin Exp Pharmacol Physiol.

[63] Banerjee T, Carrero JJ, McCulloch C, Burrows NR, Siegel KR, Morgenstern H (2021). Dietary factors and prevention: risk of end-stage kidney disease by fruit and vegetable consumption. Am J Nephrol.

[64] Dinu M, Colombini B, Pagliai G, Giangrandi I, Cesari F, Gori A (2021). Effects of vegetarian versus Mediterranean diet on kidney function: findings from the CARDIVEG study. Eur J Clin Invest.

[65] Kim H, Caulfield LE, Garcia-Larsen V, Steffen LM, Grams ME, Coresh J, Rebholz CM (2019). Plant-based diets and incident CKD and kidney function. Clin J Am Soc Nephrol.

[66] Bernier-Jean A, Prince RL, Lewis JR, Craig JC, Hodgson JM, Lim WH (2021). Dietary plant and animal protein intake and decline in estimated glomerular filtration rate among elderly women: a 10-year longitudinal cohort study. Nephrol Dial Transplant.

[67] Carrero JJ, González-Ortiz A, Avesani CM, Bakker SJL, Bellizzi V, Chauveau P (2020). Plant-based diets to manage the risks and complications of chronic kidney disease. Nat Rev Nephrol.

[68] Cupisti A, Morelli E, Meola M, Barsotti M, Barsotti G (2002). Vegetarian diet alternated with conventional low-protein diet for patients with chronic renal failure. J Ren Nutr.

[69] Soroka N, Silverberg DS, Greemland M, Birk Y, Blum M, Peer G, Iaina A (1998). Comparison of a vegetable-based (soya) and an animal-based low-protein diet in predialysis chronic renal failure patients. Nephron.

[70] Neufingerl N, Eilander A (2021). Nutrient intake and status in adults consuming plant-based diets compared to meat-eaters: a systematic review. Nutrients.

[71] Tyson CC, Lin PH, Corsino L, Batch BC, Allen J, Sapp S (2016). Short-term effects of the DASH diet in adults with moderate chronic kidney disease: a pilot feeding study. Clin Kidney J.

[72] Moorthi RN, Armstrong CL, Janda K, Ponsler-Sipes K, Asplin JR, Moe SM (2014). The effect of a diet containing 70% protein from plants on mineral metabolism and musculoskeletal health in chronic kidney disease. Am J Nephrol.

[73] St-Jules DE, Goldfarb DS, Sevick MA (2016). Nutrient non-equivalence: does restricting high-potassium plant foods help to prevent hyperkalemia in hemodialysis patients?. J Ren Nutr..

[74] González-Ortiz A, Xu H, Ramos-Acevedo S, Avesani CM, Lindholm B, Correa-Rotter R (2021). Nutritional status, hyperkalaemia and attainment of energy/protein intake targets in haemodialysis patients following plant-based diets: a longitudinal cohort study. Nephrol Dial Transplant.

[75] Te Dorsthorst RPM, Hendrikse J, Vervoorn MT, van Weperen VYH, van der Heyden MAG (2019). Review of case reports on hyperkalemia induced by dietary intake: not restricted to chronic kidney disease patients. Eur J Clin Nutr.

[76] Clase CM, Carrero JJ, Ellison DH, Grams ME, Hemmelgarn BR, Jardine MJ (2020). Potassium homeostasis and management of dyskalemia in kidney diseases: conclusions from a kidney disease: improving global outcomes (KDIGO) controversies conference. Kidney Int.

[77] Jones WL (2001). Demineralization of a wide variety of foods for the renal patient. J Ren Nutr.

[78] Scialla JJ, Anderson CA (2013). Dietary acid load: a novel nutritional target in chronic kidney disease?. Adv Chronic Kidney Dis..

[79] González-Ortiz A, Xu H, Avesani CM, Lindholm B, Cederholm T, Risérus U (2020). Plant-based diets, insulin sensitivity and inflammation in elderly men with chronic kidney disease. J Nephrol.

[80] Palmer BF, Clegg DJ (2016). Physiology and pathophysiology of potassium homeostasis. Adv Physiol Educ.

[81] Cupisti A, Kovesdy CP, D'Alessandro C, Kalantar-Zadeh K (2018). Dietary approach to recurrent or chronic hyperkalaemia in patients with decreased kidney function.

[82] Kitada M, Ogura Y, Monno I, Koya D (2018). A low-protein diet for diabetic kidney disease: its effect and molecular mechanism, an approach from animal studies. Nutrients.

